# Comment on Petre et al. Tissue Bioconcentration Pattern and Biotransformation of Per-Fluorooctanoic Acid (PFOA) in *Cyprinus carpio* (European Carp)—An Extensive In Vivo Study. *Foods* 2023, *12*, 1423

**DOI:** 10.3390/foods15071261

**Published:** 2026-04-07

**Authors:** Alison M. Zachritz, Graham F. Peaslee, Gary A. Lamberti

**Affiliations:** 1 Department of Biological Sciences and Environmental Change Initiative, University of Notre Dame, Notre Dame, IN 46556, USA; glambert@nd.edu; 2 Department of Physics and Astronomy, University of Notre Dame, Notre Dame, IN 46556, USA; gpeaslee@nd.edu

We read with interest the submission “Tissue Bioconcentration Pattern and Biotransformation of Per-Fluorooctanoic Acid (PFOA) in *Cyprinus carpio* (European Carp)—An Extensive In Vivo Study” by Petre et al. (2023) [1], because we have also studied PFAS in fish and other aquatic biota, and were pleased to see this study on PFAS concentrations measured in European Carp. We appreciated the authors’ treatment of this example of an important environmental issue. We also feel compelled to comment on their interpretation of the results.

The principle finding by Petre et al. (2023) [1] is that European (i.e., Common) Carp chronically exposed to PFOA accumulate it throughout their bodies (but especially in the gallbladder and kidneys) and that shorter-chain PFCAs (which the authors call ‘metabolites’) were also measured in all tissues. We recognize that the authors do not claim complete mineralization of PFOA in Common Carp; however, the manuscript repeatedly interprets the appearance of shorter-chain PFCAs (PFBA [C4]–PFHpA [C7]) in exposed fish as evidence of in vivo biotransformation from PFOA. We suggest that this novel mechanistic conclusion is premature without ruling out co-exposure via impurities or homologues in the dosing material, formation in the exposure system, or contamination somewhere in the sample processing workflow.

Specifically, the study reports the detection of PFBA, PFPeA, PFHxA, and PFHpA in tissues of PFOA-exposed carp and interprets the presence of these analytes as biotransformation products of PFOA. However, the data presented do not establish provenance of these short-chain PFAS, as the exposure system’s water and the fish dosing materials were not reported to be analyzed for these short-chain PFCAs. Because ample evidence exists in the literature to show that PFAS produced via Electrochemical Fluorination (ECF) routinely contain homologous impurities (of varying chain lengths) [2], the observed tissue profile may well reflect co-exposure rather than in vivo chain-shortening. We recommend tempering mechanistic conclusions unless source attribution is supported by analysis of the dosing stock and the exposure water.

Decades of published reports have described PFOA as being highly resistant to biological degradation [3,4]. In fact, PFOA is more commonly found as a terminal product of precursor breakdown (e.g., 8:2 FTOH) than metabolizable itself [5]. In addition to this research, we note an underlying fundamental thermodynamic reason why PFOA would not degrade at environmentally meaningful temperatures such as those relevant to European Carp. Quantum chemical calculations performed in aqueous medium show that initiating PFOA degradation requires a standard reduction potential of approximately +2.2 V [6], far exceeding the electrochemical range accessible to typical biological systems. By comparison, the reduction of C–Cl bonds in chlorinated compounds, which bacteria can exploit for energy, occurs at redox potentials of +250 to +600 mV, well within the range of the biological “redox ladder” [7]. Each C–F bond in PFOA requires 4–8 times more energy [6] than this, placing it well outside the range of any known biological electron carrier processes that occur in European Carp or the microorganisms they contain [8]. Other oxidative enzymatic pathways like cytochrome P450 have been shown to defluorinate pharmaceuticals and precursor PFAS (such as 6:2 FTOH) [9]. However, we are aware of no evidence that P450 enzymes can lower the energetic barriers sufficiently to break fully fluorinated perfluoroalkanes like PFOA nor any quantum investigations indicating it is thermodynamically possible. One research group has found genes for dehalogenases in a particular strain of *Acidimicrobium* bacteria, part of a microbial toolkit that could aid in PFOA breakdown, but that research remains emerging and contested [8]. It is critical that these novel findings be supported with multiple lines of evidence and plausible proposed mechanisms.

We suggest that the authors provide quantification of short-chain PFCAs (PFBA [C4] through PFHpA [C7]) in the dosing stock and in the exposure water to rule out other contamination sources for these shorter-chain PFAS. Isotope dilution is the gold standard for PFAS analysis. However, the Methods Section of Petre et al. (2023) [1] only lists a mass-labeled standard for PFOA. If mass-labeled standards for C4–C7 compounds were used, including those details would strengthen confidence in the analytical methods. Precise and reproducible quality control becomes increasingly important as known interferents for PFBA and PFPeA exist in environmental and biological samples [10]. Additional information on quantitation methods, use of pre-extraction standards and instrument blanks, and evidence that these short-chain products are absent from dosed water would increase confidence in the novel mechanism suggested.
